# Publisher Correction: Discovery of four *Noggin* genes in lampreys suggests two rounds of ancient genome duplication

**DOI:** 10.1038/s42003-020-01272-x

**Published:** 2020-09-21

**Authors:** Galina V. Ermakova, Alexander V. Kucheryavyy, Andrey G. Zaraisky, Andrey V. Bayramov

**Affiliations:** 1 Shemyakin-Ovchinnikov Institute of Bioorganic Chemistry, Russian Academy of Sciences, Moscow, 117997 Russia; 2 Severtsov Institute of Ecology and Evolution, Russian Academy of Sciences, Moscow, 119071 Russia

**Keywords:** Evolutionary developmental biology, Pattern formation

Correction to: *Communications Biology* 10.1038/s42003-020-01234-3, published online 10 September 2020.

The author affiliations in the original published version of the Article were inadvertently swapped so that the correct information for affiliation 1 was listed under affiliation 2, and vice versa. The correct author affiliations are shown below. The error has been corrected in the HTML and PDF versions of the Article.

Galina V. Ermakova^1^, Alexander V. Kucheryavyy^2^, Andrey G. Zaraisky^1^, Andrey V. Bayramov^1^

^1^Shemyakin-Ovchinnikov Institute of Bioorganic Chemistry, Russian Academy of Sciences, Moscow 117997, Russia

^2^Severtsov Institute of Ecology and Evolution, Russian Academy of Sciences, Moscow 119071, Russia

