# Supplementary material for: Discovery of four Noggin genes in lampreys suggests two rounds of ancient genome duplication
Source: Commun Biol. 2020 Sep 10;3:501. doi: 10.1038/s42003-020-01234-3 (PMC7483449; doi:10.1038/s42003-020-01234-3)
Supplement: Supplementary file 1 — Supplementary Information [file 42003_2020_1234_MOESM1_ESM.doc]

Supplementary Information for

**Discovery of four *Noggin* genes in lampreys suggests two rounds of ancient genome duplication**

FIGURES


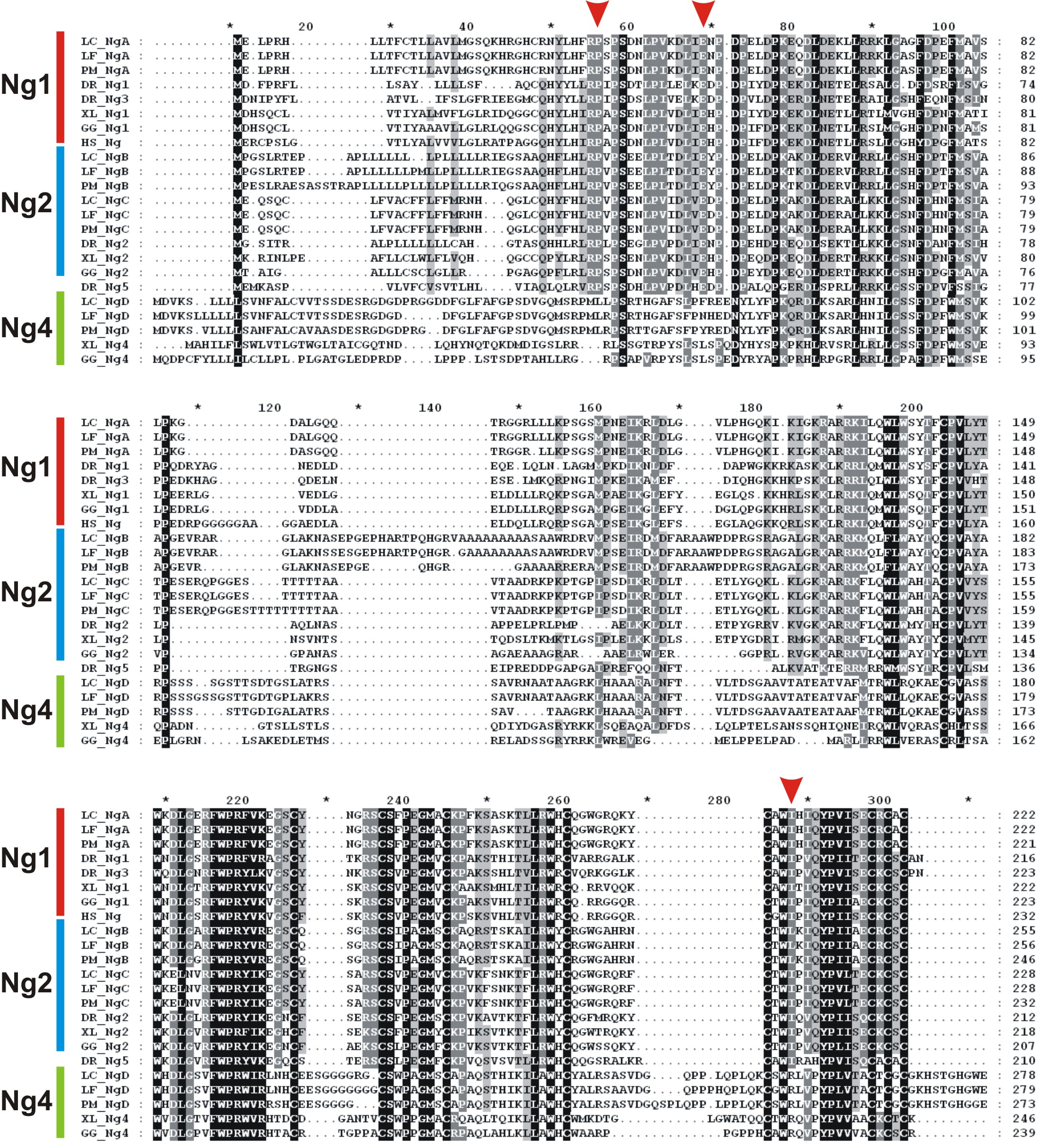


***Supplementary Figure 1.*** Comparison of the amino acid sequences of the Noggin family proteins of lampreys with the Noggin protein sequences of bony fish (*Danio rerio*, DR), amphibians (*Xenopus laevis*, XL), birds (*Gallus gallus*, GG) and Human (HS – Homo sapiens).

The Noggin protein sequences of three lampreys are presented: Arctic lamprey (*Lethenteron camtschaticum*, LC), sea lamprey (*Petromyzon marinus*, PM) and European river lamprey (*Lampetra fluviatilis*, LF).

Red arrowheads mark amino acid substitutions in positions critical for the binding of BMP ^1, 2^.


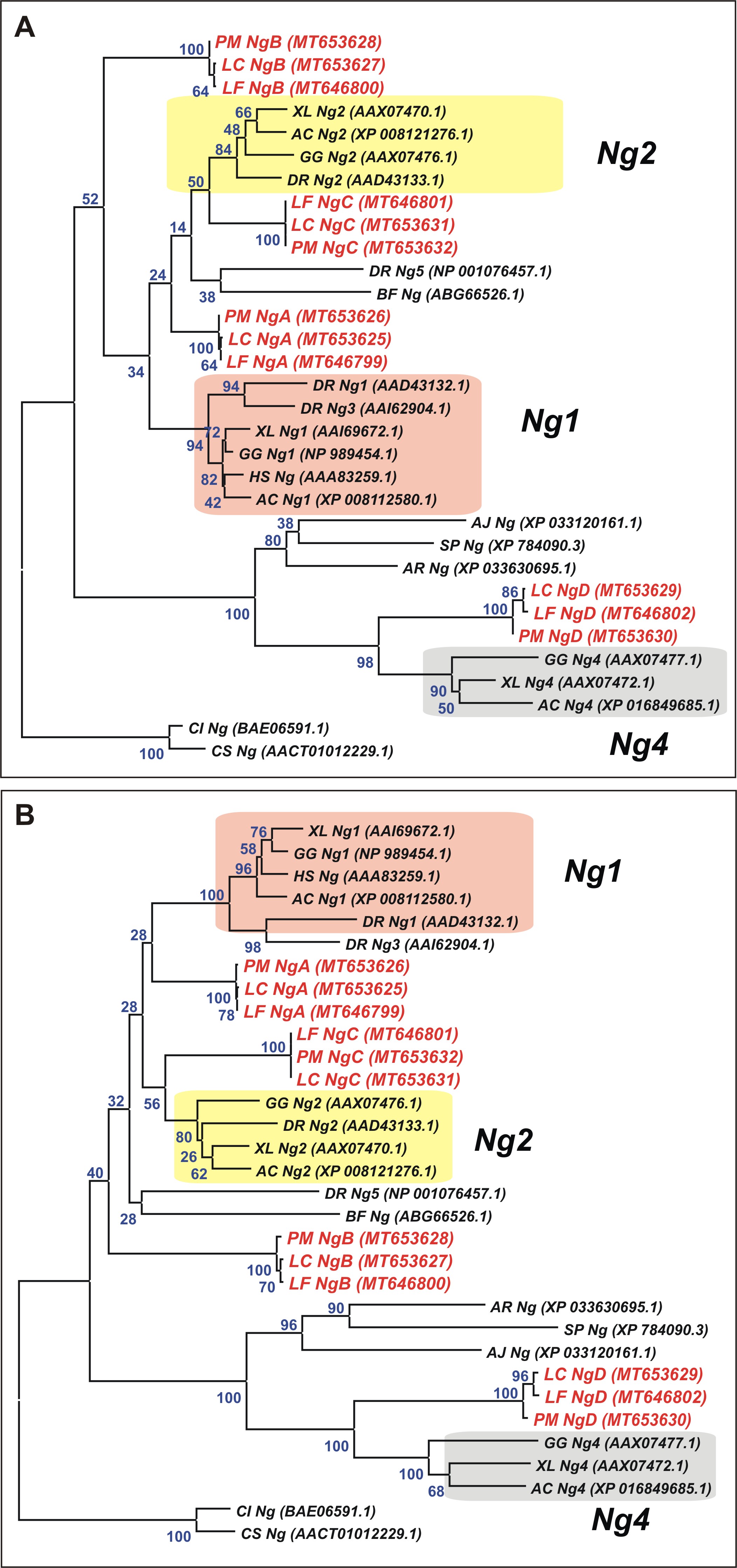


***Supplementary Figure 2.*** ML and NJ phylogenetic tree including *Chordata* and *Echinodermata* Noggins.

CS - *Ciona savygnyi*, AS- *Asterias rubens*, AJ - *Anneissia japonica*, SP - *Strongylocentrotus purpuratus*


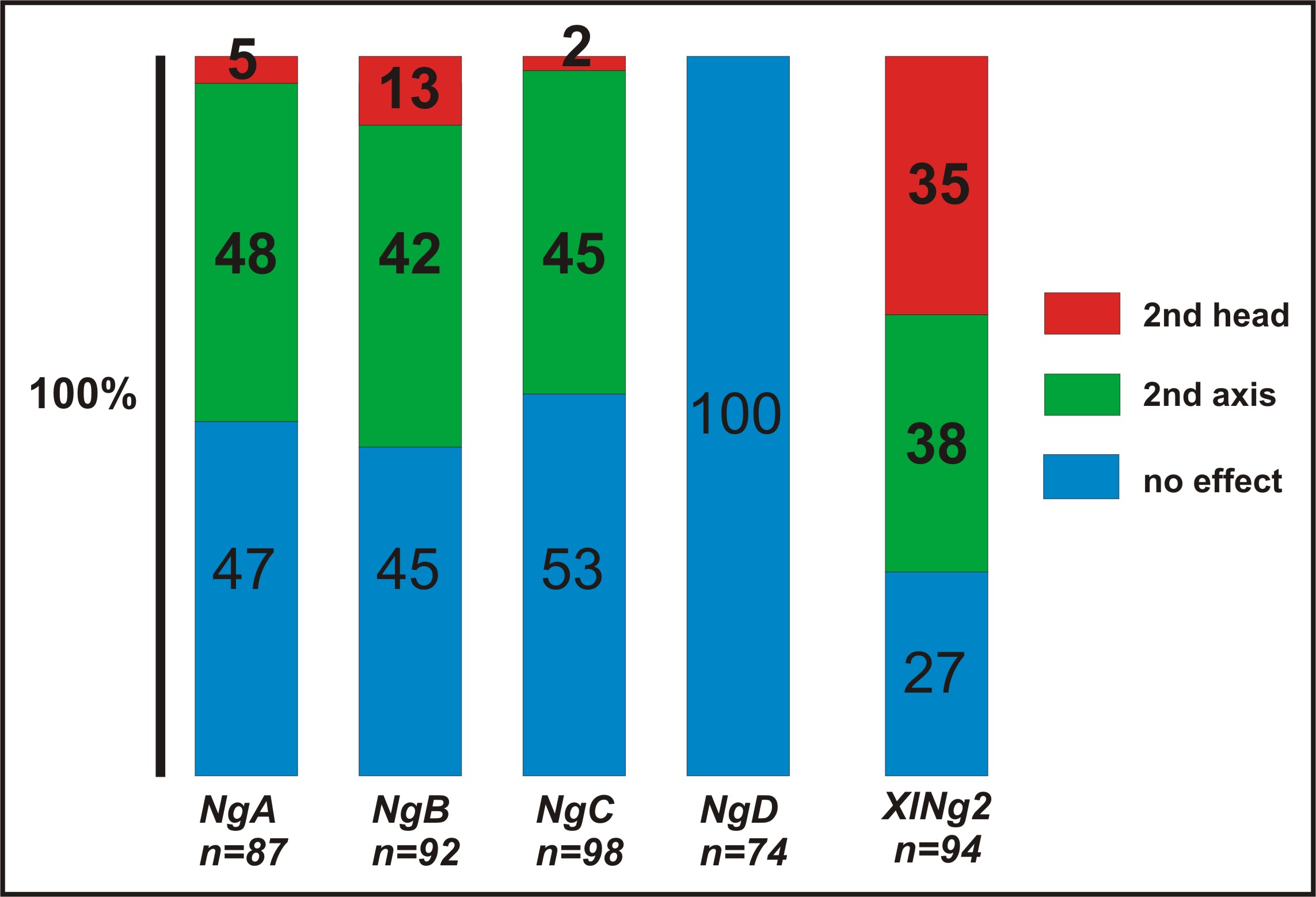


***Supplementary Figure 3.*** Percentage of secondary axis induction by lamprey *Noggins* mRNAs microinjected in *Xenopus* embryos.

**SUPPLEMENTARY Methods**

**Cloning of lamprey *Noggins* cDNAs**

Full-length cDNAs of *L. fluviatilis* *Noggin* genes were obtained by nested PCR with following pairs of primers:

*Lf_NgA_full_Frw1:* ATTGGATCCGGTGCCCGCGGCTCCATGAA;

*Lf_NgA_full_Frw2:* ATTGAATTCGCCACCATGAACTGTGGTCGTGTGGA;

*Lf_NgA_full_Rev1:* AATCTCGAGTACAGGGCTGGTCTCAG;

*Lf_NgA_full_Rev2:* AATCTCGAGTCAGCAGGCGCAGCGGCA;

*Lf_NgB_full_Frw1:* ATTGGATCCCGCGCCGAGACCATCAT;

*Lf_NgB_full_Frw2:* ATTGAATTCGCCACCATGCCGGGGTCCCTGCG;

*Lf_NgB_full_Rev1:* AATCTCGAGCCCTCGTCGTCTCAGCAG;

*Lf_NgB_full_Rev2:* AATCTCGAGTCAGCAGGAGCACCTGCACTCG;

*Lf_NgC_full_Frw1:* ATTGGATCCCGTATGCCGAACAAATGGAG;

*Lf_NgC_full_Frw2:* ATTGAATTCGCCACCATGGAGCAGTCGCAGTGTT;

*Lf_NgC_full_Rev1:* AATCTCGAGCGGATGTCCCCCCATCAGC;

*Lf_NgC_full_Rev2:* AATCTCGAGTCAGCACGAGCATTTG;

*Lf_NgD_full_Frw1:* ATTGAATTCACTTCGACGCAGCCATGGA;

*Lf_NgD_full_Frw2:* ATTGAATTCGCCACCATGGATGTGAAGAGC;

*Lf_NgD_full_Rev1:* AATCTCGAGTTTGCTGCGGGGAGATTCA;

*Lf_NgD_full_Rev2:* AATCTCGAGTCACTCCCATCCGTGTCC.

The first round of PCR (30 cycles) was done with Forward1 and Reverse1 primers. Then, the PCR mixture was diluted 1000 times and other 20 PCR cycles were performed by using Forward2 and Reverse2 primers.

**RT-PCR**

For the first strand synthesis 250 ng of total RNA extracted from each sample was reverse transcribed in 20 μl of final volume by M-MLV reverse transcriptase (Promega) in presence of 10 pmol of oligo-dT primer (Evrogen), according to the manufacturer's guidelines (Promega) (+RT sample). In parallel, same reaction was assembled in each case without adding of M-MLV reverse transcriptase (-RT control). For qPCR reaction, which was performed on DT*Prim4* (DNA Technology), 2μl of +RT and -RT solutions of each type were mixed in parallel tubes with qPCRmix-HS SYBR (x5, Evrogen), corresponding primers (5pmol each) and milli-Q water till the final volume 25 μl. A standard 40-cycle program with hot start was used; the annealing temperature was 59°C, elongation – 72°C and melting 95 °C, all lasted for 25 seconds. The PCR data were imported into Microsoft Excel and analyzed by using the ΔΔC_t_ method. The geometric mean of expression of two reference housekeeping genes: *ornithine decarboxylase* (*ODC*) and *elongation factor 1alpa* (*EF1alpha*) was used for normalization of the target genes expression levels.

The following pairs of primers designed by Primer-Blast tool on the base of full-length *L. fluviatilis* sequences were used:

*Lf_NgA_ RT-Frw1:* TGAACTGTGGTCGTGTGGAG;

*Lf_NgA_ RT-Rev1:* AGTGCAGGTAGTTGCGACAG;

*Lf_NgA_ RT-Frw2:* TGGGAGGTCGTGTTCCTTTC;

*Lf_NgA_ RT-Rev2:* TCGGAGATGACGGGGTACTG;

*Lf_NgB_ RT-Frw1:* GCTGGTACTGCCGTGGGT;

*Lf_NgB_ RT-Rev1:* ACCTGCACTCGGCTATGATG;

*Lf_NgB_ RT-Frw2:* CGCGCAACATTTCCTCCAC;

*Lf_NgB_ RT-Rev2:* CTCCTCAGCACCCTCTCGTC;

*Lf_NgC_ RT-Frw1:* ATGGAGCAGTCGCAGTGTTT;

*Lf_NgC_ RT-Rev1:* ACAGGGAGGTTTTCACTCGG;

*Lf_NgC_ RT-Frw2:* GCAGCAACTTCGACCACAAC;

*Lf_NgC_ RT-Rev2:* TGGGCTTTGGCTTCCTGTC;

*Lf_NgD_ RT-Frw1:* GATGTGAAGAGCTTGCTGCTG;

*Lf_NgD_ RT-Rev1:* GTCCGAAAGCGAAGAGACCA;

*Lf_NgD_ RT-Frw2:* ACGGCGATGACTTTGGTCTC;

*Lf_NgD_ RT-Rev2:* CCTCGTGGTTTGGAAAGCTG;

*Lf_EF1alfa-_Frw:* AGAACGTGTCTGTCAAGGATGT;

*Lf_EF1alfa_Rev:* TAGCCGGCATTGATCTGGCCA;

*Lf_ODC_Forw:* CCGTCGGTATCATCGCCAAG;

*Lf_ODC_Rev:*CGAAGAGGATGCAGTTGAAG.

**REFERENCES**

1. Eroshkin FM, Nesterenko AM, Borodulin AV, et al. Noggin4 is a long-range inhibitor of Wnt8 signalling that regulates head development in Xenopus laevis. *Sci Rep*. **6**, 23049 (2016).

2. Groppe, J. *et al.* Structural basis of BMP signalling inhibition by the cystine knot protein Noggin. *Nature* **420,** 636–42 (2002).
