# Supplementary material for: Discovery of four Noggin genes in lampreys suggests two rounds of ancient genome duplication
Source: Commun Biol. 2020 Sep 10;3:501. doi: 10.1038/s42003-020-01234-3 (PMC7483449; doi:10.1038/s42003-020-01234-3)
Supplement: Supplementary file 3 — Reporting Summary [file 42003_2020_1234_MOESM3_ESM.pdf]

nature research

Corresponding author(s): Andrey Bayramov: andrbayr@gmail.com, Andrey Zaraisky: azaraisky@yahoo.com

Last updated by author(s): Jun 24, 2020

Reporting Summary

Nature Research wishes to improve the reproducibility of the work that we publish. This form provides structure for consistency and transparency in reporting. For further information on Nature Research policies, see Authors & Referees and the Editorial Policy Checklist.

Statistics

For all statistical analyses, confirm that the following items are present in the figure legend, table legend, main text, or Methods section.

n/a	Confirmed	
☐	☒	The exact sample size (*n*) for each experimental group/condition, given as a discrete number and unit of measurement
☐	☒	A statement on whether measurements were taken from distinct samples or whether the same sample was measured repeatedly
☒	☐	The statistical test(s) used AND whether they are one- or two-sided<br>*Only common tests should be described solely by name; describe more complex techniques in the Methods section.*
☒	☐	A description of all covariates tested
☒	☐	A description of any assumptions or corrections, such as tests of normality and adjustment for multiple comparisons
☒	☐	A full description of the statistical parameters including central tendency (e.g. means) or other basic estimates (e.g. regression coefficient) AND variation (e.g. standard deviation) or associated estimates of uncertainty (e.g. confidence intervals)
☒	☐	For null hypothesis testing, the test statistic (e.g. *F*, *t*, *r*) with confidence intervals, effect sizes, degrees of freedom and *P* value noted<br>*Give P values as exact values whenever suitable.*
☒	☐	For Bayesian analysis, information on the choice of priors and Markov chain Monte Carlo settings
☒	☐	For hierarchical and complex designs, identification of the appropriate level for tests and full reporting of outcomes
☒	☐	Estimates of effect sizes (e.g. Cohen's *d*, Pearson's *r*), indicating how they were calculated

Our web collection on statistics for biologists contains articles on many of the points above.

Software and code

Policy information about availability of computer code

Data collection	SIMRBase Petromyzon marinus genome browser: https://genomes.stowers.org/organism/Petromyzon/marinus<br>Xenopus tropicalis 9.1 on JBrowser: http://www.xenbase.org/common/displayJBrowse.do?data=data/xt9_1
Data analysis	MEGA6 - Phylogenetic analysis<br>ClustalW - Sequence multiple alignment

For manuscripts utilizing custom algorithms or software that are central to the research but not yet described in published literature, software must be made available to editors/reviewers. We strongly encourage code deposition in a community repository (e.g. GitHub). See the Nature Research guidelines for submitting code & software for further information.

Data

Policy information about availability of data

All manuscripts must include a data availability statement. This statement should provide the following information, where applicable:

- Accession codes, unique identifiers, or web links for publicly available datasets
- A list of figures that have associated raw data
- A description of any restrictions on data availability

Lamprey cDNAs Noggins GenBank accession numbers:
P. marinus NogginA MT653626, NogginB MT653628, NogginC MT653632, NogginD MT653630;
L. camtchaticum NogginA MT653625, NogginB MT653627, NogginC MT653631, NogginD MT653629;
L. fluviatilis NogginA MT646799, NogginB MT646800, NogginC MT646801, NogginD MT646802.

Field-specific reporting

Please select the one below that is the best fit for your research. If you are not sure, read the appropriate sections before making your selection.

☒ Life sciences ☐ Behavioural & social sciences ☐ Ecological, evolutionary & environmental sciences

For a reference copy of the document with all sections, see nature.com/documents/nr-reporting-summary-flat.pdf

Life sciences study design

All studies must disclose on these points even when the disclosure is negative.

Sample size	For RT-PCR experiments one sample included 50 embryos at each stage
Data exclusions	No data were excludes from the analysis
Replication	Each measurement was carried out at least three times
Randomization	Embryos were collected in samples randomly
Blinding	Investigators were blinded to group allocation during data collection

Reporting for specific materials, systems and methods

We require information from authors about some types of materials, experimental systems and methods used in many studies. Here, indicate whether each material, system or method listed is relevant to your study. If you are not sure if a list item applies to your research, read the appropriate section before selecting a response.

Materials & experimental systems

n/a	Involved in the study	
☒	☐	Antibodies
☒	☐	Eukaryotic cell lines
☒	☐	Palaeontology
☐	☒	Animals and other organisms
☒	☐	Human research participants
☒	☐	Clinical data

Methods

n/a	Involved in the study	
☒	☐	ChIP-seq
☒	☐	Flow cytometry
☒	☐	MRI-based neuroimaging

Animals and other organisms

Policy information about studies involving animals; ARRIVE guidelines recommended for reporting animal research

Laboratory animals	European river lamprey Lampetra fluviatilis
Wild animals	L. fluviatilis adult lampreys were collected in the Saint Petersburg district. Embryos were obtained via artificial fertilization of eggs squeezed from pregnant females.
Field-collected samples	All the samples were collected in the laboratory
Ethics oversight	All animal experiments were performed in accordance with guidelines approved by the Shemyakin-Ovchinnikov Institute of Bioorganic Chemistry (Moscow, Russia) Animal Committee and handled in accordance with the 1986 Animals (Scientific Procedures) Act and Helsinki Declaration.

Note that full information on the approval of the study protocol must also be provided in the manuscript.
